# Sexual Assault Prevention via Bystander Intervention Using Instagram Reels as a Communication Channel: Experimental Design Study

**DOI:** 10.2196/86512

**Published:** 2026-04-28

**Authors:** Leticia Couto

**Affiliations:** 1 College of Communication, DePaul University, 14 E Jackson Blvd, Chicago, IL, 60605, United States, 1 3123621025

**Keywords:** bystander intervention, message evaluation, sexual violence prevention, social media, college students

## Abstract

**Background:**

Bystander intervention is one of the most commonly used methods to curb the sexual violence crisis on college campuses. Most universities conduct training among their student bodies to ensure students are familiar with the procedure. However, it is necessary to remind and repeat messages to audiences to underscore their importance and solidify that knowledge among populations.

**Objective:**

In this study, the author developed Instagram-type reel messages that consider multiple frameworks used to develop bystander training and programs, such as the social norms approach and bystander barriers.

**Methods:**

These messages were tested via a 4×1 experimental design study with college students at a large public university (N=157). The conditions were messages that emphasize norms (norm reinforcement condition, based on the social norms approach; n=39), messages that emphasize behavior (norm readjustment condition, based on bystander barriers; n=39), messages that underscore the discrepancy between perceived peer norm and actual behavior (combination condition, based on both the social norms approach and bystander barriers; n=39), and a control condition (n=40).

**Results:**

ANOVA analysis revealed that the norms reinforcement condition and the combination condition seemed to have the greatest impact on participants’ perceived norms and behavioral intentions. In particular, when assessing norms as the dependent variable, the reinforcement group was significantly different from the readjustment group (Δmean 0.682, SD 0.160; *P<.*001), the combination group (Δmean 0.675, SD 0.160; *P<.*001), and the control group (Δmean 0.432, SD 0.159; *P*=.04). For behavioral intentions to engage in bystander intervention, the reinforcement group was significantly different from the control group (Δmean 0.217, SD 0.073; *P*=.02), and the combination group was significantly different from the control group as well (Δmean 0.221, SD 0.073; *P=.*02).

**Conclusions:**

The findings indicate that these messages could work in conjunction with training as a way to underscore the importance of bystander intervention behavior. It also highlights the role that Instagram reels can play in the prevention of sexual violence on college campuses.

## Introduction

### Background

The issue of sexual assault on college campuses in the United States is well documented [1,2]. Traditional training procedures have been used to address this concern, most prominently bystander intervention training efforts [3]. These trainings are often delivered in person or online [4]. However, considering the social media usage among college-aged adults being high (particularly Instagram at 78%) [5], it is necessary to explore mobile health (mHealth) efforts as a complementary way to enhance these trainings.

mHealth can be defined as “a medical and public health practice supported by mobile devices, such as mobile phones, patient monitoring devices, personal digital assistants, and other wireless devices” [6]. The success of mHealth strategies varies depending on the subject matter of the intervention and how the success was measured [7,8]. They can also vary based on the delivery mode, such as text messages and gamification efforts [7]. Considering this context, using social media platforms to share prosocial behavior messaging tailored for a specific population can fall under the mHealth umbrella, particularly if the social media effort does not exist in a vacuum and is combined with existing training on college campuses.

The first step to develop a proper message to be shared on social media, particularly on platforms such as Instagram and its reels, is a strong tailoring effort in terms of the message [9]. In Couto [10], potential messages were piloted and assessed as Instagram reels addressing engagement in bystander intervention behavior using a social norms approach [11] in combination with Burn’s [12] bystander intervention barriers, specifically the barrier that refers to failure to take responsibility. As defined in Burn [12], the failure to take responsibility barrier refers to people’s reluctance to engage in bystander intervention because of the belief that others will do so instead. Conversely, the social norms approach suggests that people are likely to engage in a behavior if others are doing so as well [11]. Using those 2 approaches to develop 3 different messages, Couto [10] explored the feasibility and credibility of these initial messages, which were subsequently adapted for this study, as described in the *Methods* section.

However, as this study evaluates the effects of these messages, we must not rely solely on the frameworks used to develop the messages but also consider how prosocial behavior can be propagated. An often-used theoretical approach to predict prosocial behavior is the integrative model of behavioral prediction [13], which is an expansion of the theory of planned behavior [14] that includes factors that can influence not only the intentions to engage in a behavior but also environmental factors, such as media consumption [15]. Instagram reels exposure, thus, can be considered one of these background factors. The theory of planned behavior strictly suggests that the attitudes, norms, and efficacy constructs described in the framework refer to the behavior they are predicting specifically [14], whereas the integrative model of behavioral prediction allows for more flexibility [13].

Many studies have explored how to increase bystander intervention among different programs (eg, [16,17]), and a number of them use the integrative model of behavioral prediction or the theory of planned behavior to explain how people come to the decision to engage in bystander intervention (eg, [18,19]), as these theories focus on attitudes toward the behavior, norms in relation to the behavior, and efficacy in engaging in the behavior. However, this study focuses on the novelty of Instagram reels as a tool not commonly used for mHealth efforts, particularly in bystander intervention campaigns, while also considering Burn’s [12] bystander barriers. It is important to note that Burn [12] refers specifically to barriers associated with the steps of the bystander intervention process, rather than to broader systemic barriers such as gender norms [20].

Instagram is a global social media platform widely used among college-aged individuals [21]. Instagram reels, in particular, are short videos that can be scrolled through or shared via Instagram stories. Instagram reels have increasingly been used by health care professionals, particularly in efforts to improve health literacy [22,23]. However, there is a gap between content created by health care professionals and content developed in alignment with a broader prevention approach, such as those implemented by universities in the United States to address sexual violence on their campuses.

### Current Study

In this study, Instagram reel-type messages were tested in terms of incentivizing bystander intervention among college students. These messages have been extensively piloted [10] and were tested in a 4×1 experimental design, where each condition represents one of the main frameworks used to study bystander intervention behavior: the social norms approach [24], bystander intervention barriers [12], a combination of both, and a control. The study seeks to explore which of these conditions increases perceived norms, efficacy, attitudes, intentions to intervene, and intentions to take responsibility.

The following hypotheses were developed based on the literature discussed here and the theoretical frameworks used:

- H1: Participants in the norm readjustment condition will have higher intentions to take responsibility than those in the norm reinforcement condition.
- H2: Both norm readjustment and norm reinforcement conditions score higher regarding the intention to take responsibility, perceived peer norm, attitudes, efficacy, and intentions to engage in bystander intervention when compared to the control condition.

The following research question (RQ) was developed in consideration of the lack of evidence regarding the possible effects of combining the bystander barriers and social norms approaches to develop a bystander intervention message, and how it would perform compared to single-theory approaches:

- RQ1: Will the norm readjustment condition significantly differ from the norm reinforcement condition in (1) perceived peer norms, (2) self-efficacy, (3) attitudes, and (4) intentions?

Considering that the combination condition is theoretically exploratory, the following RQs were developed:

- RQ2: Does combining readjustment and reinforcement messages improve participants’ intentions to engage in bystander behavior?
- RQ3: Are (1) perceived peer norms, (2) self-efficacy, (3) attitudes, and (4) intentions higher in the combination condition than in the other experimental conditions?

## Methods

### Procedures and Participants

Participants were recruited by requesting a list of student emails from the Registrar’s Office of the university where the study took place, targeting a group of 2000 undergraduate students, 500 from each class standing (ie, first-year students, sophomores, juniors, and seniors). The request was made on September 22, 2023. The 2000 students were emailed, and a total of 157 participants responded, resulting in a 7.5% response rate. In the sample, 61.3% (n=68) of the participants identified as women, 94.5% (n=104) identified as cisgender, and 69.1% (n=76) identified as heterosexual. In terms of race and ethnicity, 73% (n=81) identified as White, followed by 10.8% (n=12) as Asian American and 5.6% (n=6) as a race not listed in the options; 60% (n=66) indicated that they are not Hispanic/Latino/a. The participants were also predominantly middle class in terms of socioeconomic status (n=81, 73%), and the plurality of the participants were first-year students (n=39, 35.1%). The age range was 18 to 29 years (mean 19.97, SD 1.81 years). Considering that normative messaging is tailored to specific groups in mind, the sample is appropriate as the recruitment strategy of requesting emails from the Registrar’s Office can yield a sample that reflects the student body, even though it is a convenient sample. For a full breakdown of the demographic characteristics, see Multimedia Appendix 1.

Participants received an email with a link to the study. They first had to respond to screeners concerning whether they were university students currently in the United States; were between the ages of 18 and 29 years; and consent to participate in the study by signing the consent form, which is the first material they saw when they are redirected from their emails.

The study design is a posttest-only four (norm readjustment, norm reinforcement, norm readjustment and reinforcement, and control) by one experiment showing variations of an Instagram reel that is 20 seconds long concerning bystander intervention behavior. This Instagram reel effort has been rigorously tested quantitatively and qualitatively in terms of credibility, reliability, validity, and perceived message effect with a different sample with similar characteristics [10]. Table 1 shows a breakdown of each condition, its theoretical framework, and an example message. Messages were developed using data from the population (different sample) that focused on normative behavior. The visuals included a party video, and the audio featured uplifting music. The messages are slightly different from the ones tested in Couto [10] to account for the feedback participants shared on credibility. Participants responded to close-ended survey items about the message’s production and credibility, as well as open-ended items. Multimedia Appendix 1 provides a list of links to examples of the messages tested in this study.

**Table 1.** Description of conditions, theoretical frameworks, and example messages of study investigating the effect of different messaging in the format of Instagram reels on college students’ intentions to engage in bystander intervention behavior in 4×1 experimental design study.

Condition	Theory	Example message
Readjustment	Barriers	BE AN ACTIVE BYSTANDER Only 48% of (students) have told someone that their drink had been drugged (based on the response of 249 university students in Spring 2023) IT’S ON YOU! Be part of the group that helps!
Reinforcement	Social norms	BE AN ACTIVE BYSTANDER More than 97% of (students) believe that their friends would make sure another friend was ok if they seem to be in an uncomfortable situation at a party (based on the response of 249 university students in Spring 2023) IT’S ON YOU! Be part of the group that helps!
Combination	Barriers + social norms	BE AN ACTIVE BYSTANDER More than 95% of (students) believe that their friends would discourage a friend from getting someone drunk to have sex with them, but only 55% of (students) have done so (based on the response of 249 university students in Spring 2023) IT’S ON YOU! Be part of the group that helps!
Control	N/A^a^	BE SAFE AT YOUR NEXT PARTY! At parties, make sure you know where the nearest exit is in case of an emergency. You never know when you might have to bounce! PLAN AHEAD! Safe partying is more fun!

a N/A: not applicable.

### Ethical Considerations

The institutional review board that granted approval to this study deemed the study exempt from further review (20080‐001). The institutional review board is housed at Washington State University. Participants received an online informed consent form before completing the study and had to agree to its terms before participating in the study. Participants had the choice to provide their email in a Qualtrics form unlinked from the study questionnaire to enter a draw for the chance to win 1 of 4 US $20 Walmart gift cards or one $100 Tango gift card for their vendor of choice. The data were collected anonymously, and it was not possible to link the email data used for incentive distribution to the study data.

### Measures

#### Bystander Intervention Barriers: Failure to Take Responsibility

Burn [12] developed bystander intervention barrier items that are widely used in the field. Participants were asked to indicate how much they disagree (1) or agree (5) with these items on a Likert-type scale. Example items include “I am less likely to intervene to reduce a person’s risk of sexual assault if I think she/he made choices that increased their risk” and “If a person is dressed provocatively, or acts provocatively, I feel less responsible for preventing others from taking sexual advantage of them.” This is a subscale from a scale with 5 factors that reflect the 5 types of bystander intervention barriers identified by Burn [12]. This subscale has 8 items (Cronbach α=0.85). In its original study [12], the Cronbach α was 0.85.

#### Bystander Intervention Efficacy

To address bystander intervention efficacy, the items that the University of New Hampshire adapted from Banyard [25] in short form [26] were used. The scale, originally called the Bystander Efficacy Scale, contained 4 items preceded by the following statement: “Please read each of the following behaviors. Indicate in the column Confidence how confident you are that you could do them. Rate your degree of confidence by recording a whole number from 0 to 100 using the scale given below,” with a scale ranging from 0 (cannot do) to 100 (very certain). Example items include “Get help and resources for a friend who tells me they have been raped” and “Do something if I see a woman surrounded by a group of men at a party who looks very uncomfortable” (Cronbach α=0.74). In the original study [25], the Cronbach α was 0.87.

#### Bystander Intervention Attitudes

An adapted version of McMahon’s [27] bystander intervention attitude scale was used, called the Bystander Attitude Scale, Revised, which was adapted from Banyard [25]. Participants were presented with items such as “Say something to my friend who is taking a drunk person back to his/her room at a party” and asked to answer how much they agree (5) or disagree (1) that the actions described in the items are appropriate (Cronbach α=0.80). Cronbach α for the scale was not reported in McMahon [27].

#### Bystander Intervention Perceived Norms

Three items were adapted from Hust et al [28]. Participants were asked to indicate how much they agree (5) or disagree (1) with statements related to perceived norms. Items include the stem “most students at my university would,” followed by items such as “make sure their friend is ok if they see him/her in an uncomfortable sexual situation at a party” and “discourage a friend who said they planned to get someone drunk to have sex” (Cronbach α=0.79). In Hust et al [28], the Cronbach α was 0.69.

#### Intentions to Intervene in an Alcohol-Involved Sexual Assault Situation

Three items were adapted from Hust et al [28]. Participants were asked to indicate how much they agree (5) or disagree (1) with statements related to intentions. Items include the stem “I would” followed by items such as “make sure their friend is ok if they see him/her in an uncomfortable sexual situation at a party” and “discourage a friend who said they planned to get someone drunk to have sex” (Cronbach *α*=0.78). In Hust et al [28], the Cronbach α was 0.75.

## Results

### Statistical Analysis

#### Power Analysis

To define the size of the sample necessary to conduct the analyses described below, a power analysis was conducted using a medium-to-large effect size (0.15 for a regression, 0.3 for an ANOVA [29], a power of 0.8, and a significance level of .05). The power analysis indicated that for a 4×1 ANOVA, a sample of 128 participants (32 people per group) is necessary. Therefore, the current sample size is appropriate.

#### One-Way ANOVA

In an ANOVA, there are three assumptions: (1) data normality, which can be checked by investigating whether kurtosis and skewness fall within the range of [−2,2], as well as ensuring there are more than 30 individuals per group; (2) the independence of the sample, which is verified via data collection; and (3) the homogeneity of the samples, which can be ensured by having evenly sized groups or by using the Levene test [30]. Considering all groups are generally equal, all assumptions have been met.

#### Missing Data Analysis

To check the type of missing data in the dataset, Little’s [31] MCAR (missing completely at random) analysis was conducted. Little’s MCAR analysis indicated that the data are not MCAR. In this event, an expectation-maximization imputation method was used to fill in the missing data by considering other variable values when creating values for the missing data. This method is more sophisticated than mean imputation [32], making the sample more robust.

### Hypothesis and Research Questions Testing

Hypothesis 1 suggested that participants in the norm readjustment condition would have higher intentions to take responsibility than those in the norm reinforcement condition. This hypothesis was rejected as the ANOVA test indicated that there were no differences between the 4 experimental groups (*F*_3,153_=0.506; *P*=.68).

The second hypothesis purported that both the readjustment and reinforcement conditions would score higher in terms of the intention to take responsibility, perceived peer norms, attitudes, efficacy, and intentions to engage in bystander intervention when compared to the control condition. This hypothesis was partially supported. Post hoc results indicated that participants in the reinforcement condition indicated higher perceived peer norms (Δmean 0.432, SD 0.159; *P*=.04) and intentions (Δmean 0.217, SD 0.073; *P*=.02) compared to participants in the control condition. Participants in the readjustment condition did not indicate significantly higher means for norms (Δmean 0.250, SD 0.159; *P=.*40), attitudes (Δmean 0.183, SD 0.083; *P=.*13), efficacy (Δmean 0.059, SD 0.123; *P=.*96), intention to take responsibility (Δmean 0.120, SD 0.160; *P=.*88), or intentions to intervene (Δmean 0.164, SD 0.073; *P*=.11) compared to the control.

RQ 1 explored the differences between the readjustment and reinforcement conditions in terms of norms, efficacy, attitudes, and intentions to intervene. The ANOVA omnibus test indicated that there was a difference between all 4 conditions in terms of norms (*F*_3,153_=7.965; *P*<.001) and intentions (*F*_3,153_=4.095; *P*=.008). The post hoc Tukey honestly significant difference (HSD) test indicated that the significant difference in norms came from participants in the reinforcement condition, indicating higher perceived peer norms than participants in the readjustment (Δmean 0.682, SD 0.160; *P<.*001), combination (Δmean 0.675, SD 0.160; *P<.*001), and control conditions (Δmean 0.432, SD 0.159; *P=.*04). In terms of intentions to intervene, the post hoc Tukey HSD test revealed that participants in the reinforcement condition indicated higher intentions to intervene compared to participants in the control condition (Δmean .217, SD=.073; *P=.*02), but there was no statistically significant difference from the readjustment condition (Δmean 0.053, SD 0.073; *P*=.89).

The second RQ considers if combining the information from the reinforcement and readjustment conditions (the combination condition) would improve intentions to intervene compared to the other conditions. As mentioned earlier, the ANOVA omnibus test revealed that there are significant differences between the 4 groups in terms of intentions to intervene. The post hoc Tukey HSD test shows that the combination condition has a significantly higher intentions to intervene mean compared to the control group (Δmean 0.221, SD=0.073; *P=.*02). However, this is also true for the reinforcement conditions, and there are no significant differences between the reinforcement and combination conditions (Δmean 0.004, SD 0.073; *P*>.99). Thus, there seems to be no clear improvement when comparing the combination condition to other conditions in terms of intentions to intervene.

The final RQ inquired if the combination condition would be higher in terms of intentions, attitudes, efficacy, and norms than the other conditions. From previous ANOVA results discussed here, it is known that there is a difference between conditions only in terms of norms and intentions. The post hoc Tukey HSD test shows that the combination condition shows a significant improvement on the control condition in terms of intention, but it is significantly lower than the reinforcement condition in terms of norms (Δmean 0.675, SD 0.160; *P*<.001). Tables2^4^ provide the descriptive information for each condition and each outcome, a summary of the ANOVA results, and a summary of the post hoc Tukey HSD test results, respectively.

**Table 2.** Descriptives from each ANOVA condition in the study investigating the effect of different messaging in the format of Instagram reels on college students’ intentions to engage in bystander intervention behavior in 4×1 experimental design study.

Dependent variable and condition		Participants, n	Mean (SD)	SE
Norm				
Reinforcement		39	4.5354 (0.54484)	0.08724
Readjustment		39	3.8535 (0.76213)	0.12204
Combination		39	3.8602 (0.79889)	0.12793
Control		40	4.1036 (0.70049)	0.11076
Total		157	4.0882 (0.75416)	0.06019
Attitudes				
Reinforcement		39	4.4892 (0.29431)	0.04713
Readjustment		39	4.5203 (0.34800)	0.05572
Combination		39	4.5128 (0.33215)	0.05319
Control		40	4.3371 (0.47873)	0.07569
Total		157	4.4640 (0.37476)	0.02991
Efficacy				
Reinforcement		39	4.4377 (0.52525)	0.08411
Readjustment		39	4.4186 (0.46870)	0.07505
Combination		39	4.1344 (0.66494)	0.10648
Control		40	4.3594 (0.51387)	0.08125
Total		157	4.3377 (0.55590)	0.04437
Barrier				
Reinforcement		39	1.9418 (0.64281)	0.10293
Readjustment		39	1.9969 (0.77568)	0.12421
Combination		39	2.0882 (0.66285)	0.10614
Control		40	2.1166 (0.75949)	0.12009
Total		157	2.0364 (0.70950)	0.05662
Intentions				
Reinforcement		39	4.9594 (0.08613)	0.01379
Readjustment		39	4.9067 (0.24222)	0.03879
Combination		39	4.9631 (0.16172)	0.02590
Control		40	4.7424 (0.56404)	0.08918
Total		157	4.8919 (0.33196)	0.02649

**Table 3.** ANOVA results in the study investigating the effect of different messaging in the format of Instagram reels on college students’ intentions to engage in bystander intervention behavior in 4×1 experimental design study.

Outcomes		SS^a^	*df*	MS^b^	*F* test (*df*)	ω^2^	*P* value
Norm							
Between groups		11.985	3	3.995	7.965 (3, 153)	0.117	<.001
Within groups		76.742	153	0.502	—^c^	—	—
Total		88.727	156	—	—	—	—
Attitudes							
Between groups		0.885	3	0.295	2.148 (3, 153)	0.021	.097
Within groups		21.024	153	0.137	—	—	—
Total		21.909	156	—	—	—	—
Efficacy							
Between groups		2.276	3	0.759	2.528 (3, 153)	0.028	.06
Within groups		45.931	153	0.300	—	—	—
Total		48.208	156	—	—	—	—
Barrier							
Between groups		0.772	3	0.257	0.506 (3, 153)	−0.010	.68
Within groups		77.758	153	0.508	—	—	—
Total		78.530	156	—	—	—	—
Intentions							
Between groups		1.278	3	0.426	4.095 (3, 153)	0.056	.008
Within groups		15.913	153	0.104	—	—	—
Total		17.190	156	—	—	—	—

a SS: sum of squares.

b MS: mean square.

c Not applicable.

**Table 4.** Post hoc Tukey honestly significant difference (HSD) results of ANOVA in the study investigating the effect of different messaging in the format of Instagram reels on college students’ intentions to engage in bystander intervention behavior in 4×1 experimental design study.

Dependent variable	(A) Condition	(B) Conditions	ΔMean (A–B)	SE	*P* value
Norm	Reinforcement	Readjustment^a^	0.68193	0.16038	<.001
		Combination^a^	0.67521	0.16038	<.001
		Control^a^	0.43183	0.15938	.04
	Readjustment	Reinforcement^a^	−0.68193	0.16038	<.001
		Combination	−0.00672	0.16038	>.99
		Control	−0.25010	0.15938	.40
	Combination	Reinforcement^a^	−0.67521	0.16038	<.001
		Readjustment	0.00672	0.16038	>.99
		Control	−0.24338	0.15938	.42
Attitudes	Reinforcement	Readjustment	−0.03111	0.08394	.98
		Combination	−0.02368	0.08394	.99
		Control	0.15207	0.08342	.27
	Readjustment	Reinforcement	0.03111	0.08394	.98
		Combination	0.00742	0.08394	>.99
		Control	0.18318	0.08342	.13
	Combination	Reinforcement	0.02368	0.08394	.99
		Readjustment	−0.00742	0.08394	>.99
		Control	0.17575	0.08342	.16
Efficacy	Reinforcement	Readjustment	0.01909	0.12408	.99
		Combination	0.30332	0.12408	.07
		Control	0.07829	0.12330	.92
	Readjustment	Reinforcement	−0.01909	0.12408	.99
		Combination	0.28423	0.12408	.11
		Control	0.05920	0.12330	.96
	Combination	Reinforcement	−0.30332	0.12408	.07
		Readjustment	−0.28423	0.12408	.11
		Control	−0.22503	0.12330	.27
Barrier	Reinforcement	Readjustment	−0.05509	0.16144	.99
		Combination	−0.14640	0.16144	.80
		Control	−0.17479	0.16043	.70
	Readjustment	Reinforcement	0.05509	0.16144	.99
		Combination	−0.09131	0.16144	.94
		Control	−0.11970	0.16043	.88
	Combination	Reinforcement	0.14640	0.16144	.80
		Readjustment	0.09131	0.16144	.94
		Control	−0.02839	0.16043	.998
Intentions	Reinforcement	Readjustment	0.05270	0.07303	.89
		Combination	−0.00364	0.07303	>.99
		Control^a^	0.21700	0.07257	.02
	Readjustment	Reinforcement	−0.05270	0.07303	.89
		Combination	−0.05634	0.07303	.87
		Control	0.16430	0.07257	.11
	Combination	Reinforcement	0.00364	0.07303	>.99
		Readjustment	0.05634	0.07303	.87
		Control^a^	0.22064	0.07257	.02

a *P*≤.05.

## Discussion

### Principal Findings

The results indicated that the reinforcement condition seemed to have the greatest impact on the outcome variables compared to all other 3 conditions. Perceived peer norms were significantly higher among participants who were exposed to the reinforcement condition compared to all 3 conditions, and they were significantly higher in terms of intentions to intervene when compared to the control. The only other condition to show significant results was the combination condition, in which intentions to intervene were significantly higher compared to participants in the control group.

This study underscores the importance of the social norms approach when properly researched and applied. The social norms approach is commonly used as a framework for the study of reducing sexual violence on college campuses [24]. Although bystander intervention behavior exists in a different context from other prosocial behaviors that are practiced to decrease sexual violence, the importance of norms remains.

Given the lack of effect on the failure-to-take-responsibility barrier and the extremely low mean scores indicating that participants did not perceive this barrier as applicable to them, the way this barrier is conceptualized should be reconsidered. Failure to take responsibility is one of 5 barriers to engaging in bystander intervention [12]. However, it seems that these barriers are studied and considered in a linear manner, as if overcoming the first barrier opens the doors for the next step, and so on. The findings of this study challenge this assumption. Failure to take responsibility appears to be disapproved of by this population, and it was an overall nonfactor in terms of being affected by the conditions. One should consider the possibility of social desirability, but considering the responses of participants, it may reflect a reluctance to take responsibility, but rather a lack of efficacy. Further investigation is needed to determine the extent to which failure to take responsibility translates into a lack of efficacy to perform what the participants perceive as the appropriate action. Failure to take responsibility may be more closely related to a bystander’s comfort level than to the belief that it is not their problem and, therefore, they will not intervene.

The conditions did not have an effect on efficacy. However, considering the size of the sample and the significance level of the omnibus test on efficacy, it is possible a larger sample would show significant effects. Although promising, efficacy is difficult to communicate in such short messages. Even in the presence of significant results, it would be inappropriate to expect college students to develop self-efficacy skills in relation to bystander intervention with only exposure to a message. Indeed, the goal of this study is not to provide a “magic bullet” but to create a tool that can be used in conjunction with the many trainings offered on college campuses across the United States [33].

In terms of theory, the findings build upon the social norms approach by showing that pluralistic ignorance can be addressed with normative messaging [24]. However, much of this study focused on establishing that bystander intervention is a unique behavior, in that pluralistic ignorance could be detrimental given the presence of the failure-to-take-responsibility barrier. Norms, however, appeared to play a greater role than the barrier, suggesting that the traditional approach to social normative messaging development may apply to bystander intervention behavior. In fact, the underwhelming role of the barrier could explain why the combination condition affected only the intentions condition. Although this was the case, the result was still noteworthy. It shows that in other paradoxical behaviors—such as bystander intervention—where perceived norms can be a detractor instead of stimulus and normative-only messaging is not enough, combining the 2 approaches could be beneficial. There is a need to explore other behaviors that function in a similar way.

This need also extends to existing studies that explore ways to promote bystander intervention behavior among college students. Although there are many studies that explore the effects of and on norms of bystander intervention trainings (eg, [11,34]), this study appears to be the first study to examine norms directly related to bystander intervention, rather than relying on proxies such as rape myth acceptance [34]. Additionally, this study is, to the author’s knowledge, one of the first attempts to understand the bystander barriers empirically. Much of Burn’s [12] work is based on a collective understanding of the bystander intervention literature. However, once it is established that these barriers exist when examining other studies, it is necessary to examine them in the context of how these barriers present themselves in messaging tailored to address bystander intervention behavior.

Furthermore, one major aspect of this study is assessing the feasibility of Instagram reels as a way to support sexual violence prevention campaigns on college campuses. As mentioned, health care workers are already using such platforms to create health-related content [22]. Incorporating tailored messaging into a new channel through a holistic health campaign appears to be the next step in meeting audiences where they are for health literacy efforts.

With these results in mind, it is also necessary to consider next steps to expand this effort. Replication studies are needed to ensure the effect is consistent. Replication should occur not only within the same population over longitudinal waves but also across different populations (in this case, college campuses). Thus, the replication effort has two components: one addressing the depth of the message (longitudinal effort) and the other addressing the breadth of the message (multiple college campuses efforts).

There is also a need for field experiments. Although results are promising, it is not possible to determine how this message would perform if it organically appeared as an advertisement on Instagram reels. A study is needed in which participants are allowed to view the content organically in a laboratory setting, followed by an assessment of how the broader college population perceives the message on their actual Instagram accounts. In a similar vein, it is necessary to test the message in different contexts and assess if this alters its effects. This can be conducted both in a replication study and in laboratory settings, focusing on trust and credibility concerns.

Finally, the results suggest that the operationalization of failure to take responsibility may be too complex to be captured in studies as it is right now. Further research is needed to investigate the underlying causes of reluctance to help others. This research would benefit particularly from qualitative efforts.

### Limitations

Although many steps were taken to mitigate the limitations of this study, there are still limitations to be considered. First, the study was not conducted in a laboratory setting. This was a calculated risk to increase external validity. However, it is necessary to consider that some participants may not have watched the messages as attentively as they would have in a laboratory setting. In contrast, one element of external validity that could be improved is the inclusion of visual cues that indicate this is supposed to be an Instagram reel. Although the portrait orientation video is one possible cue, there are other elements that could have been included.

Another limitation is that, although experimental, this was a cross-sectional, posttest-only study. The results may be promising, but it is necessary to also assess longitudinal designs. Thus, no long-term effects can be established for this message intervention, and considering that the intent is multiple exposures to the message when presented organically on social media, an assessment of multiple exposure effects is also necessary.

Finally, the power analysis conducted for this study considered a medium-to-large effect size. However, it is possible that larger sample sizes could have detected more minute relationships with smaller effect sizes. More investigation is needed with larger samples. This can also be affected by the relatively high proportion of data (approximately 30%); therefore, the results should be interpreted cautiously since expectation-maximization imputation was used.

### Conclusions

In this study, different theoretical frameworks that can be used individually or in combination to develop messages that encourage bystander intervention behavior in sexual violence situations on college environments were explored. The messages were designed with the intent to be an Instagram reel message, as this platform is popular within the intended audience of young adult college students [35,36], leading to a wide reach. Additionally, it is worth noting that this message would work in conjunction with other university efforts to address sexual violence prevention and bystander intervention behavior. Many universities across the United States have required bystander intervention training for first-year students (. Green Dot, in particular, is used in more than 500 universities in the United States [33].

In this study, the author explored the role of dual-theory message design in addressing bystander intervention on one specific college campus. The results underscore the importance of fully researched and tested normative messaging. There are many research implications, from the role of dual-theory approaches in the message development of health communication messages to the importance of mixed methods as a methodology. Although some limitations exist, future research focusing on the breadth and depth of the messages may address many of these limitations.

## Supplementary material

10.2196/86512 Multimedia Appendix 1 Link to video of message example.

## Abbreviations

HSD: honestly significant difference
MCAR: missing completely at random
mHealth: mobile health
RQ: research question
