# Supplementary material for: Sexual Assault Prevention via Bystander Intervention Using Instagram Reels as a Communication Channel: Experimental Design Study
Source: JMIR Form Res. 2026 Apr 28;10:e86512. doi: 10.2196/86512 (PMC13124031; doi:10.2196/86512)
Supplement: Multimedia Appendix 1 [file formative-v10-e86512-s001.docx]

**Link to message Norm 1 (example for reference):** https://www.canva.com/design/DAFueKr0uLE/ggt5HHhP1St2RQhrOuBDqQ/watch?utm_content=DAFueKr0uLE&utm_campaign=designshare&utm_medium=link2&utm_source=uniquelinks&utlId=h46ed7932c1
